# Supplementary figures and images for: Synergy between the small intrinsically disordered protein Hsp12 and trehalose sustain viability after severe desiccation
Source: eLife. 2018 Jul 16;7:e38337. doi: 10.7554/eLife.38337 (PMC6054528; doi:10.7554/eLife.38337)

**Figure 4B – source data**

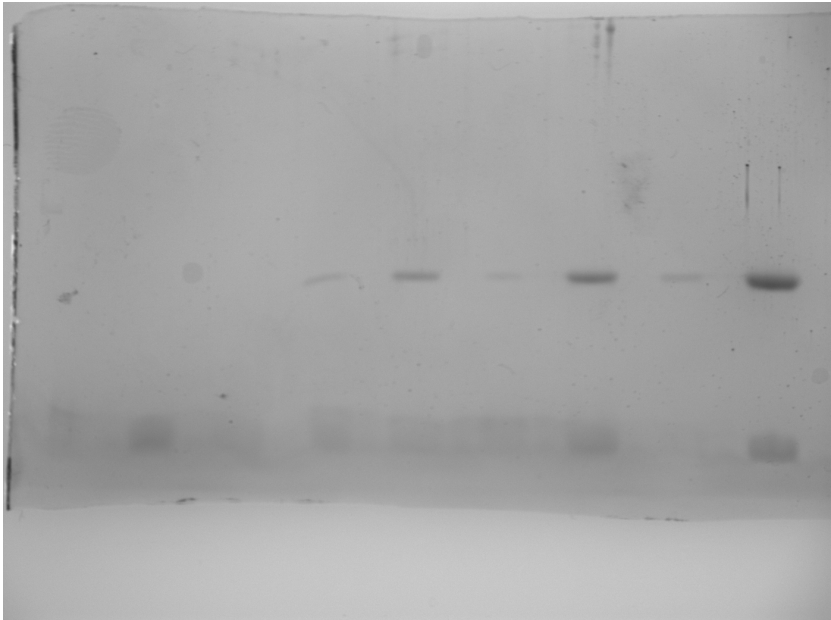

Supplement: Figure 4—source data 1. [file elife-38337-fig4-data1.pdf]

**Figure 5D – source data**

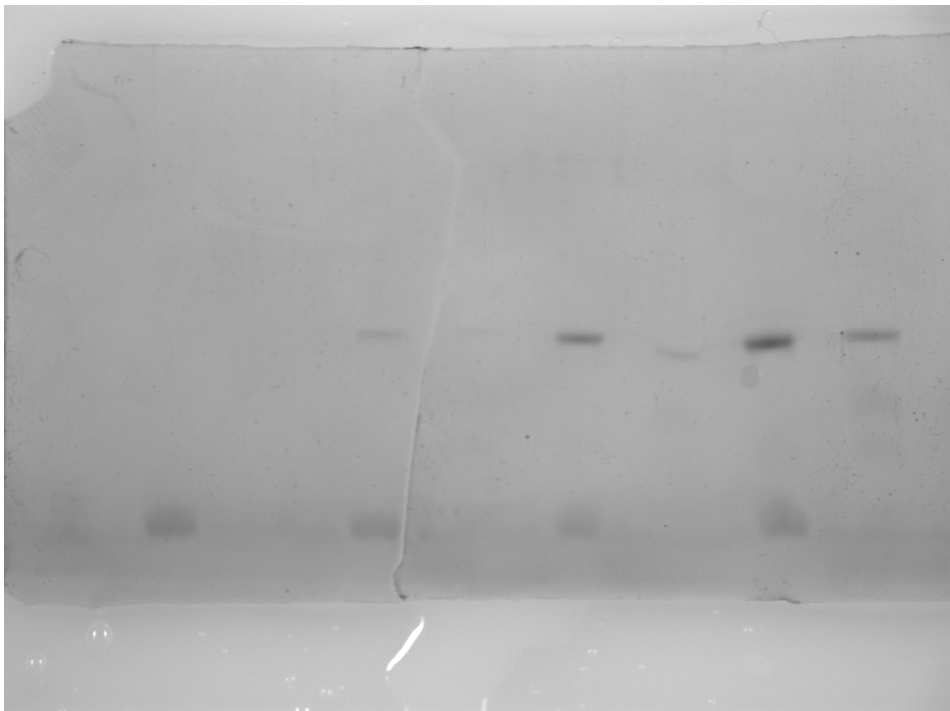

Supplement: Figure 5—source data 2. [file elife-38337-fig5-data2.pdf]

**Figure 5E – source data**

**Stf2 alone**

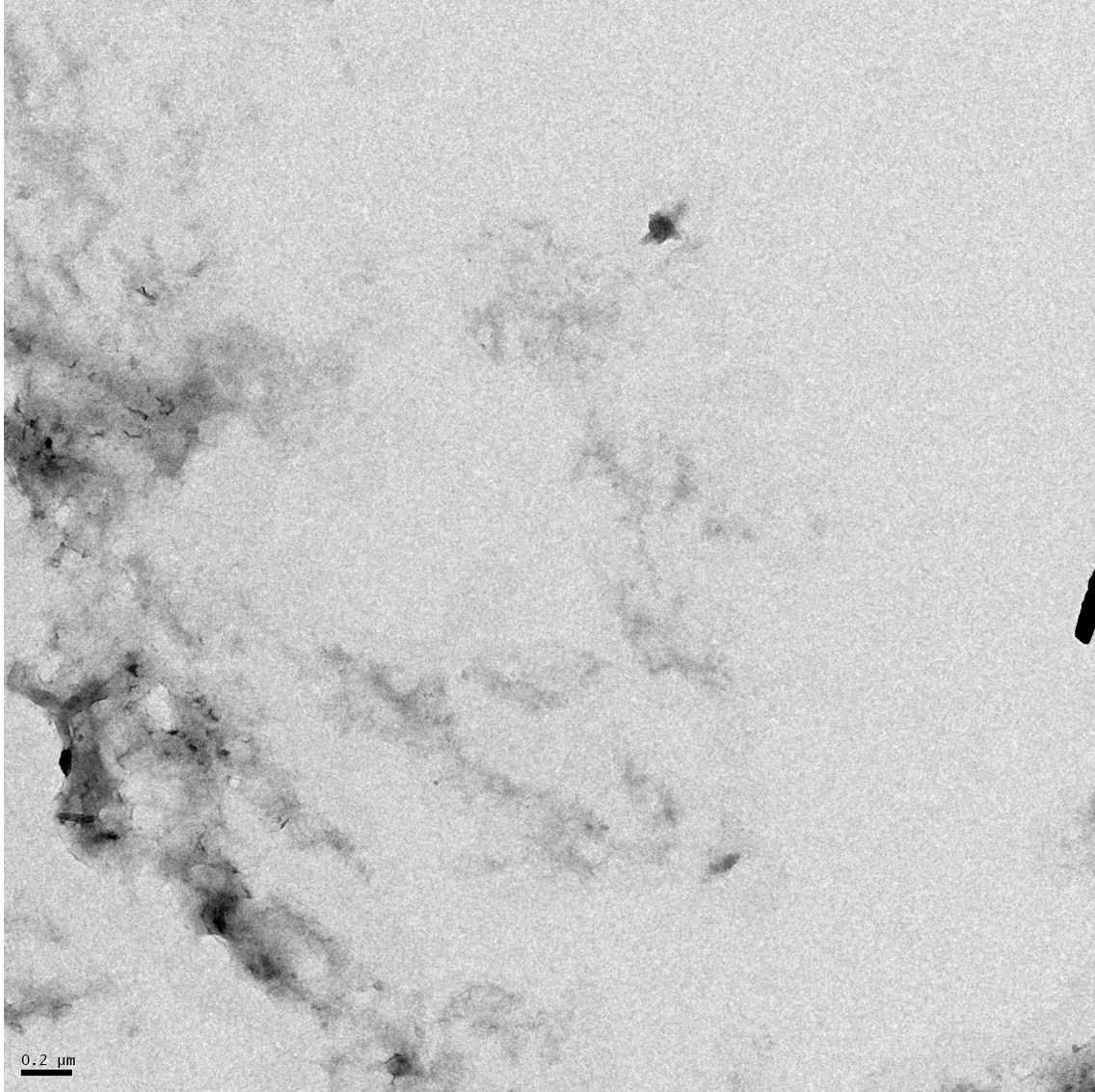

Stf2 alone

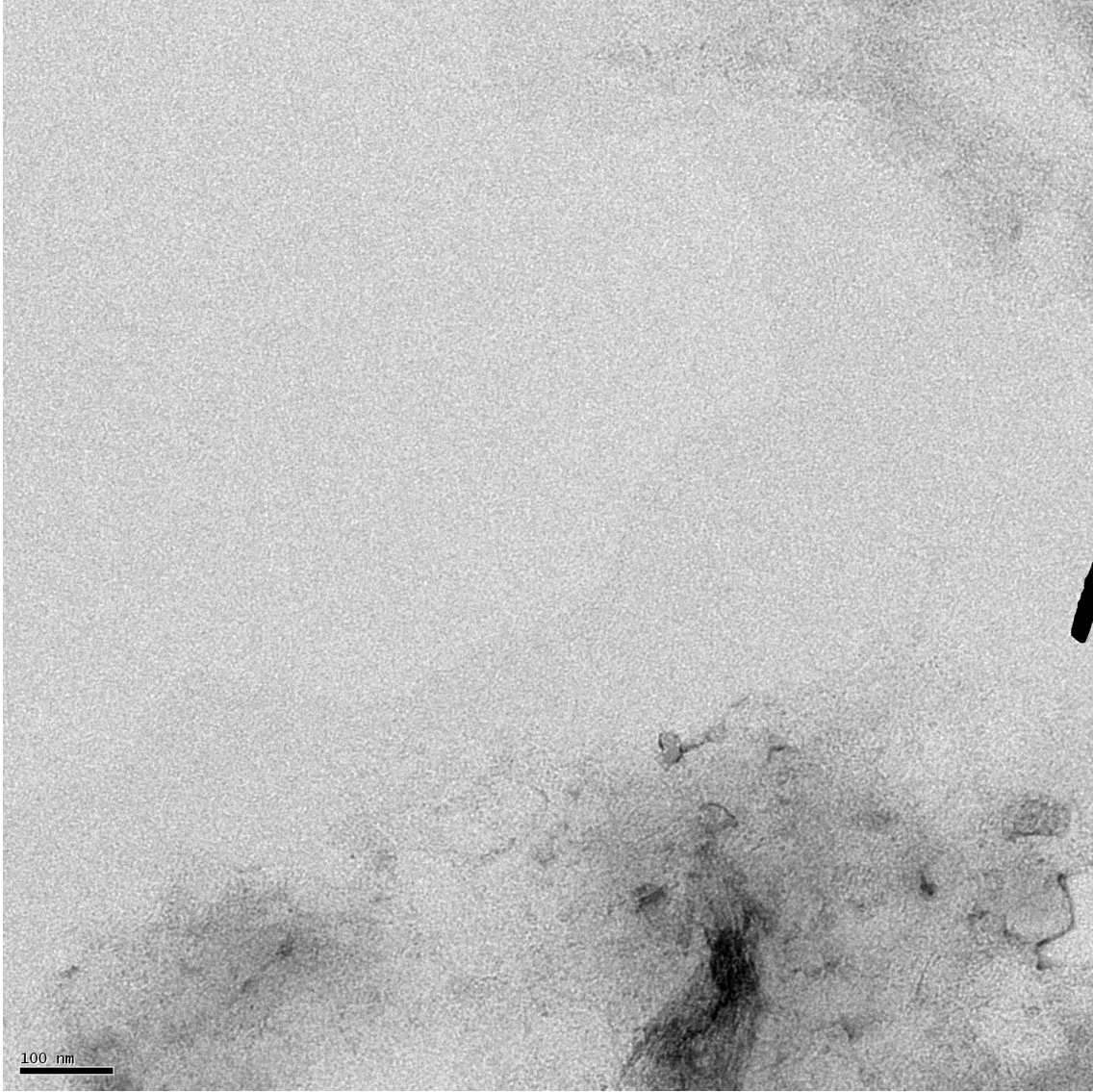

## Lipids alone

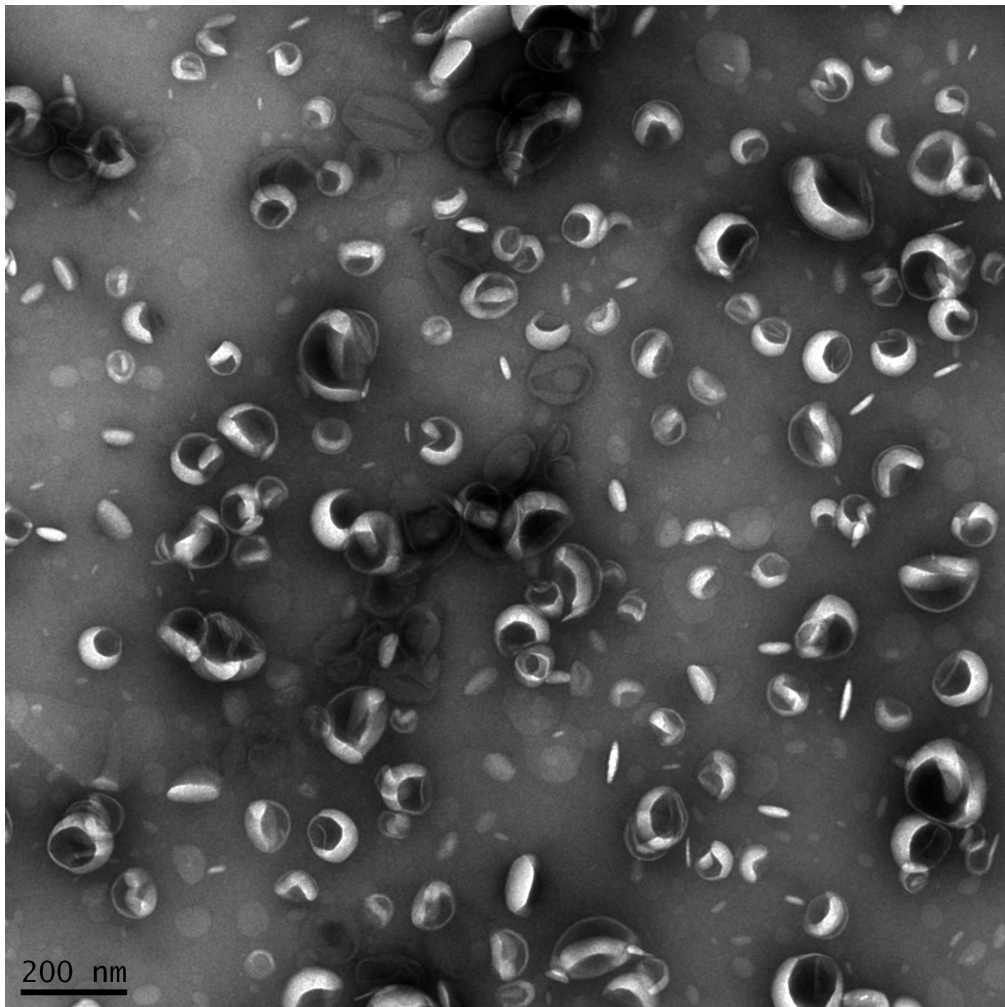

## Lipids alone

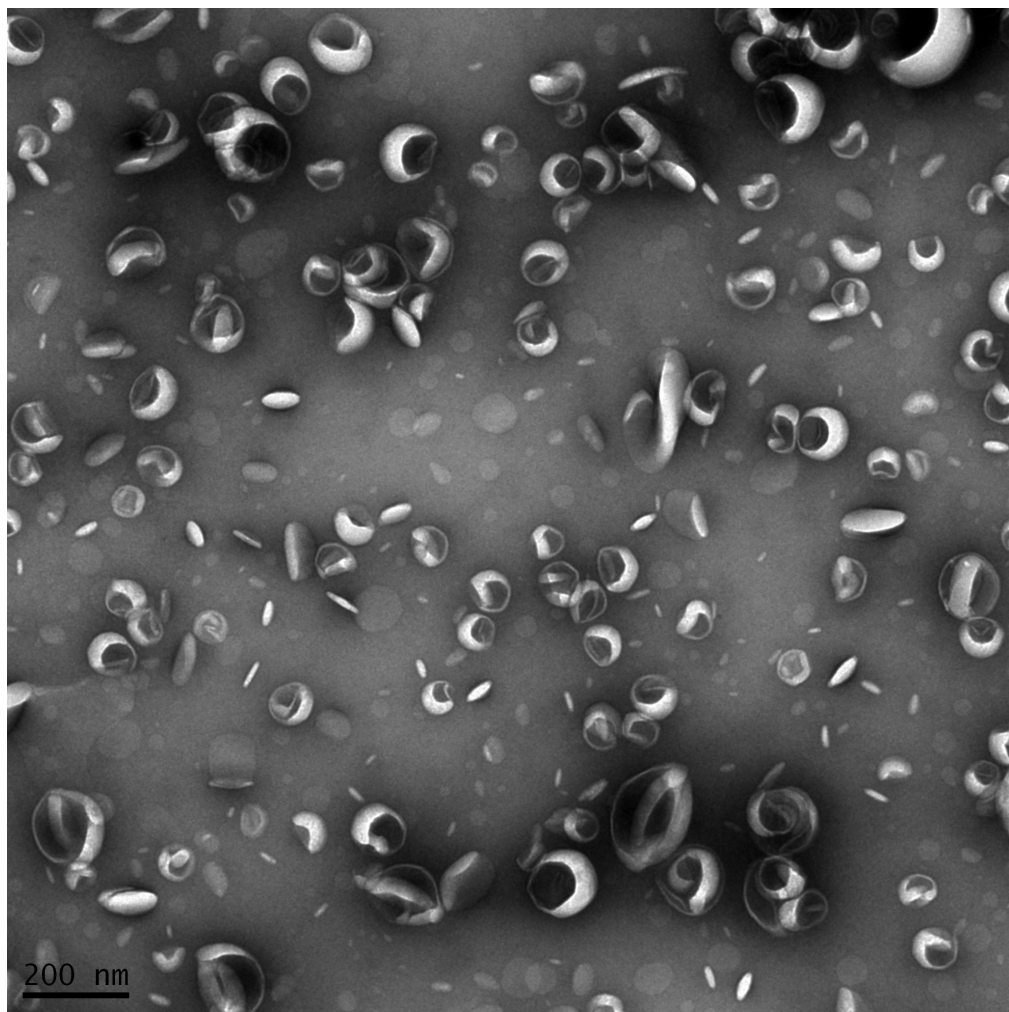

Lipids + Stf2

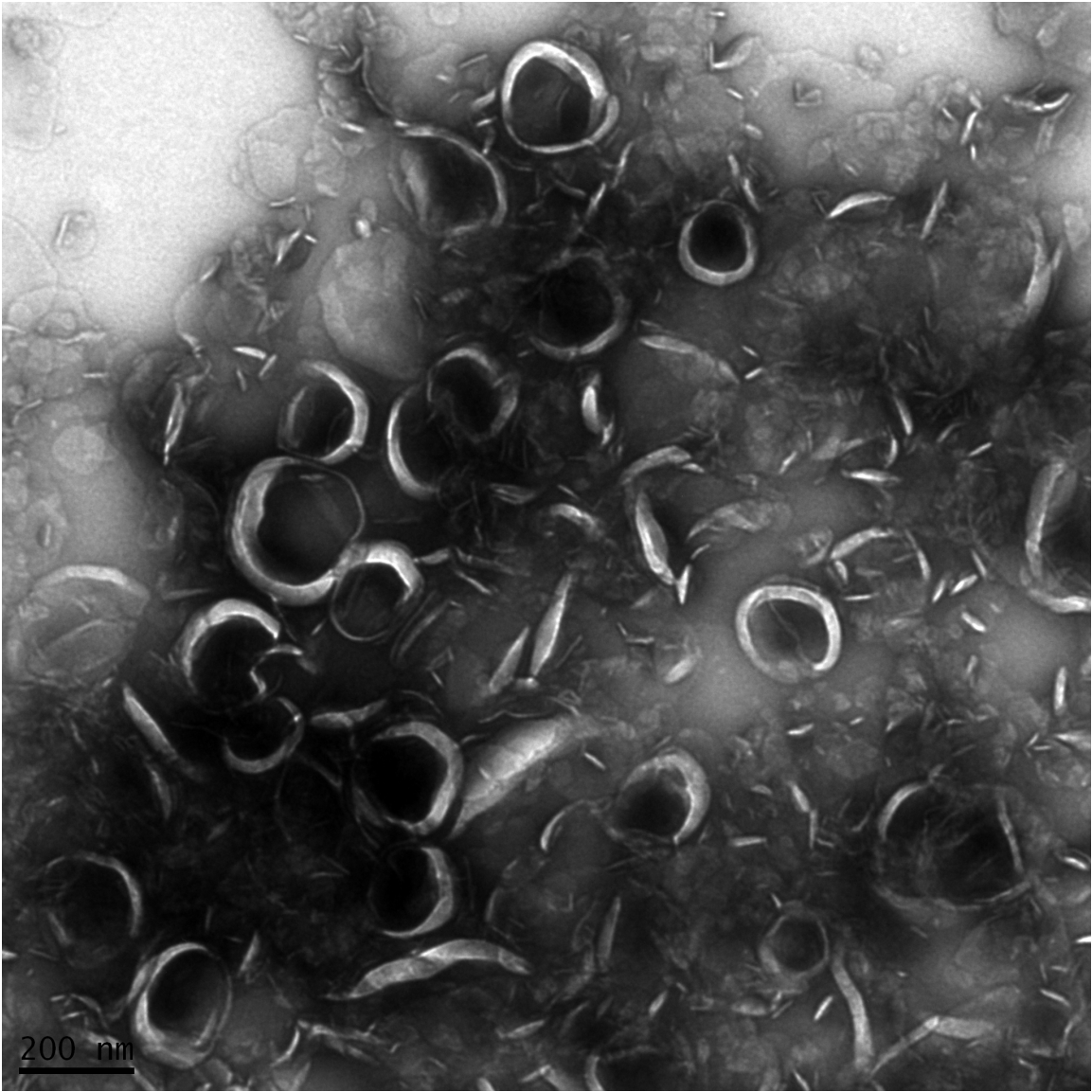

**Lipids + Stf2**

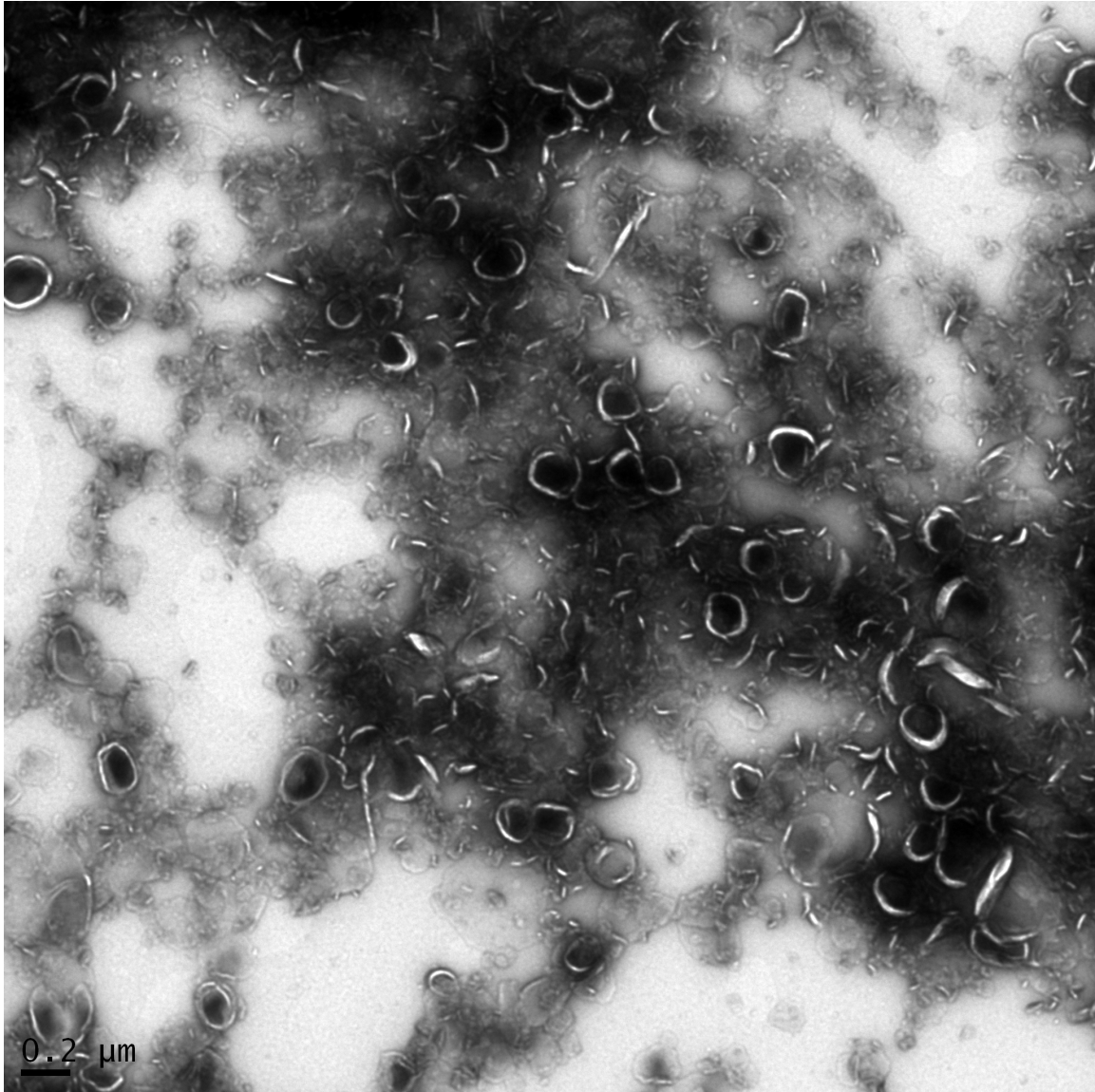

Supplement: Figure 5—source data 3. [file elife-38337-fig5-data3.pdf]

Figure Supplement 1. Trehalose and Hsp12 heat protection

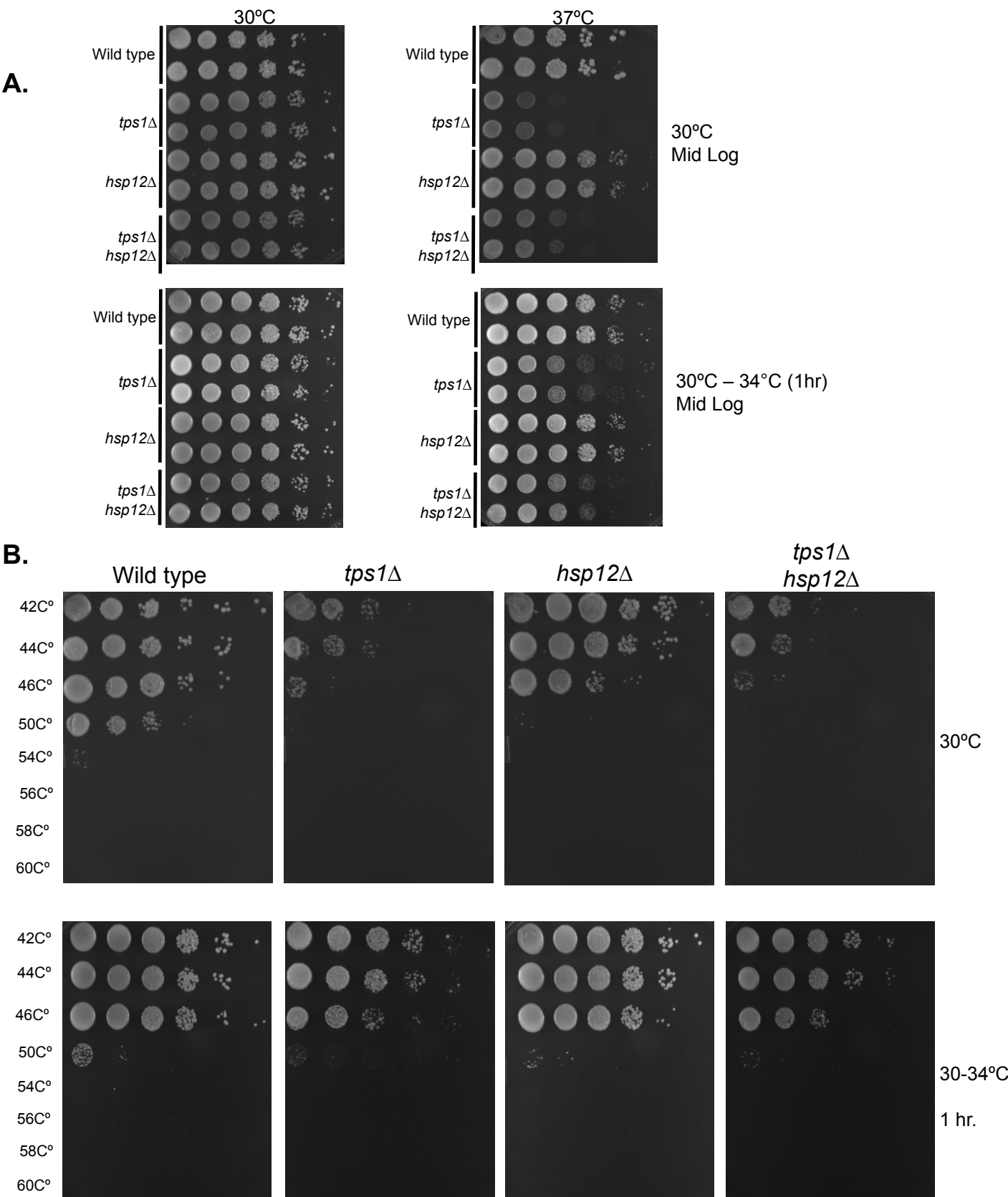

Supplement: Supplementary file 1. — Hsp12 and Stf2 are bacterial derived protein preps. Gel stained with Coomassie Blue demonstrates purity of protein preps. [file elife-38337-supp1.pdf]
