# Supplementary material for: Synergy between the small intrinsically disordered protein Hsp12 and trehalose sustain viability after severe desiccation
Source: eLife. 2018 Jul 16;7:e38337. doi: 10.7554/eLife.38337 (PMC6054528; doi:10.7554/eLife.38337)

Figure 4C – source data

Hsp12 alone

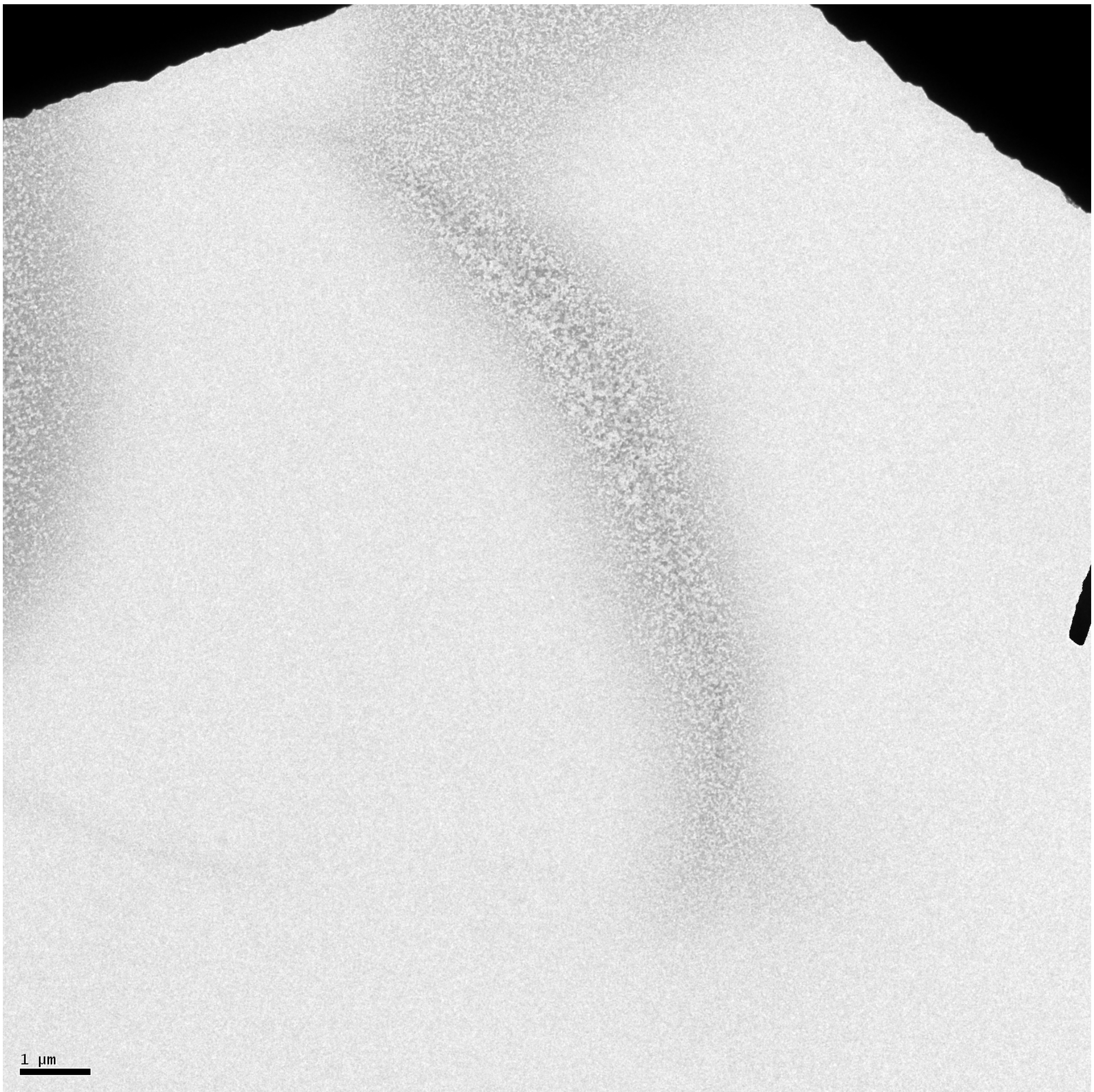

Hsp12 alone

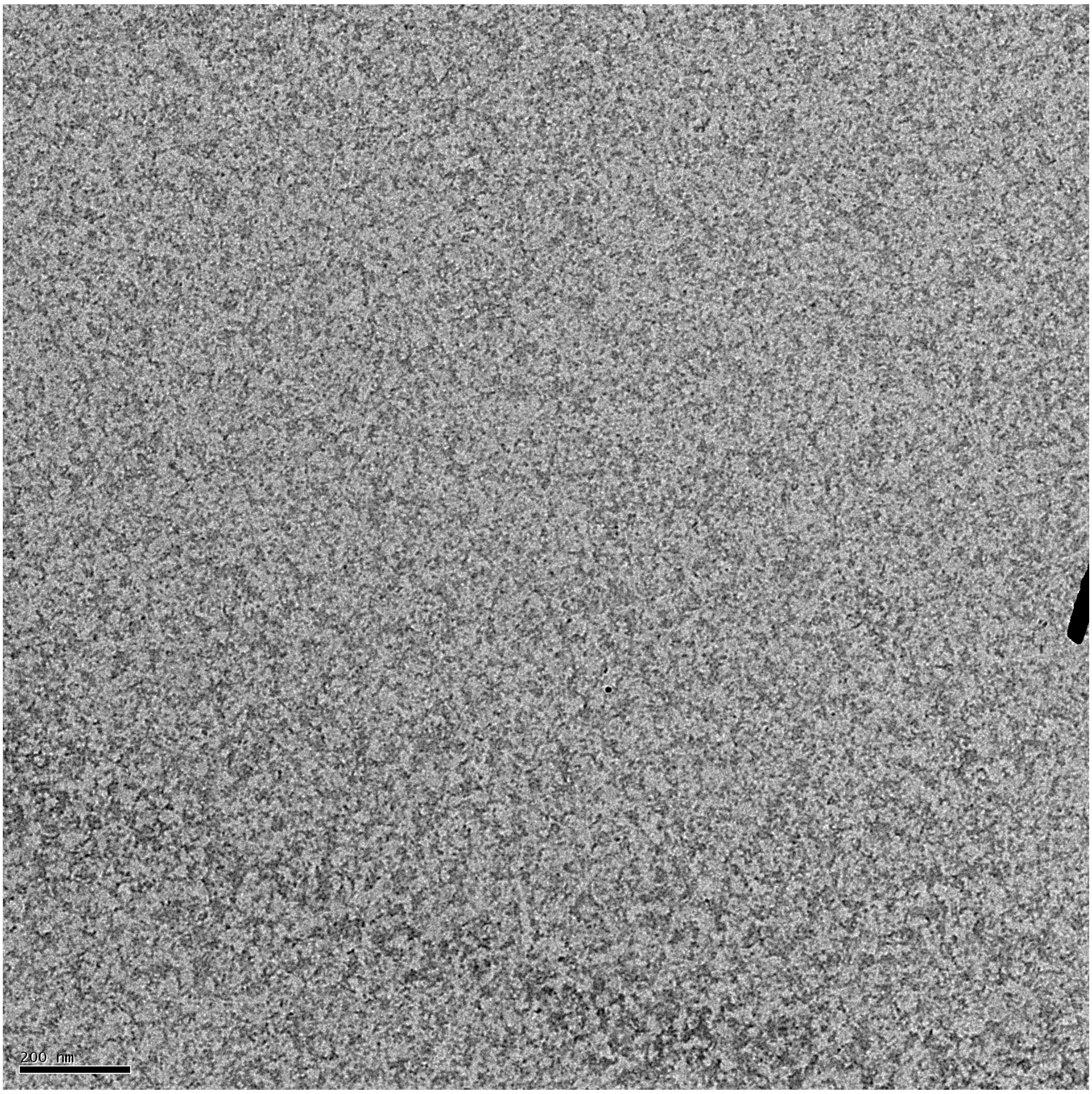

Lipids alone

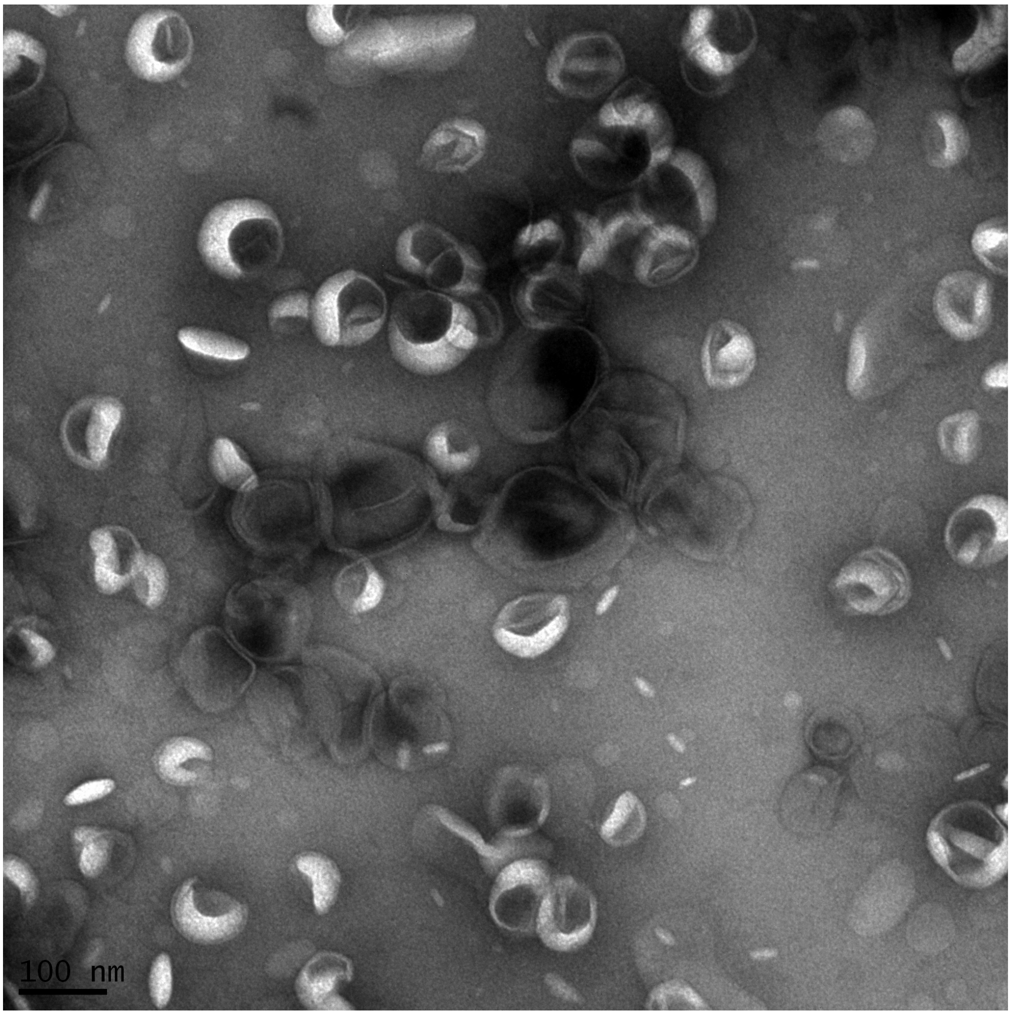

Lipids alone

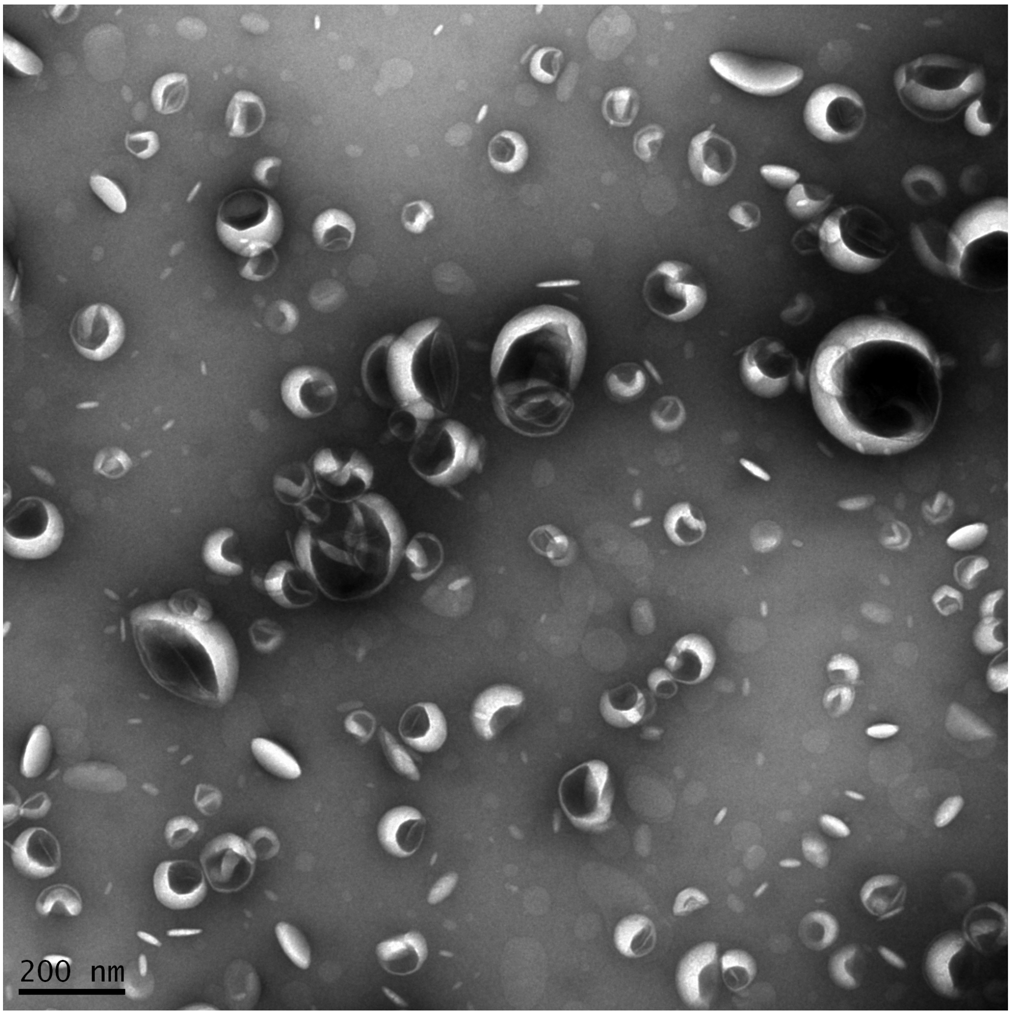

Lipids + Hsp12

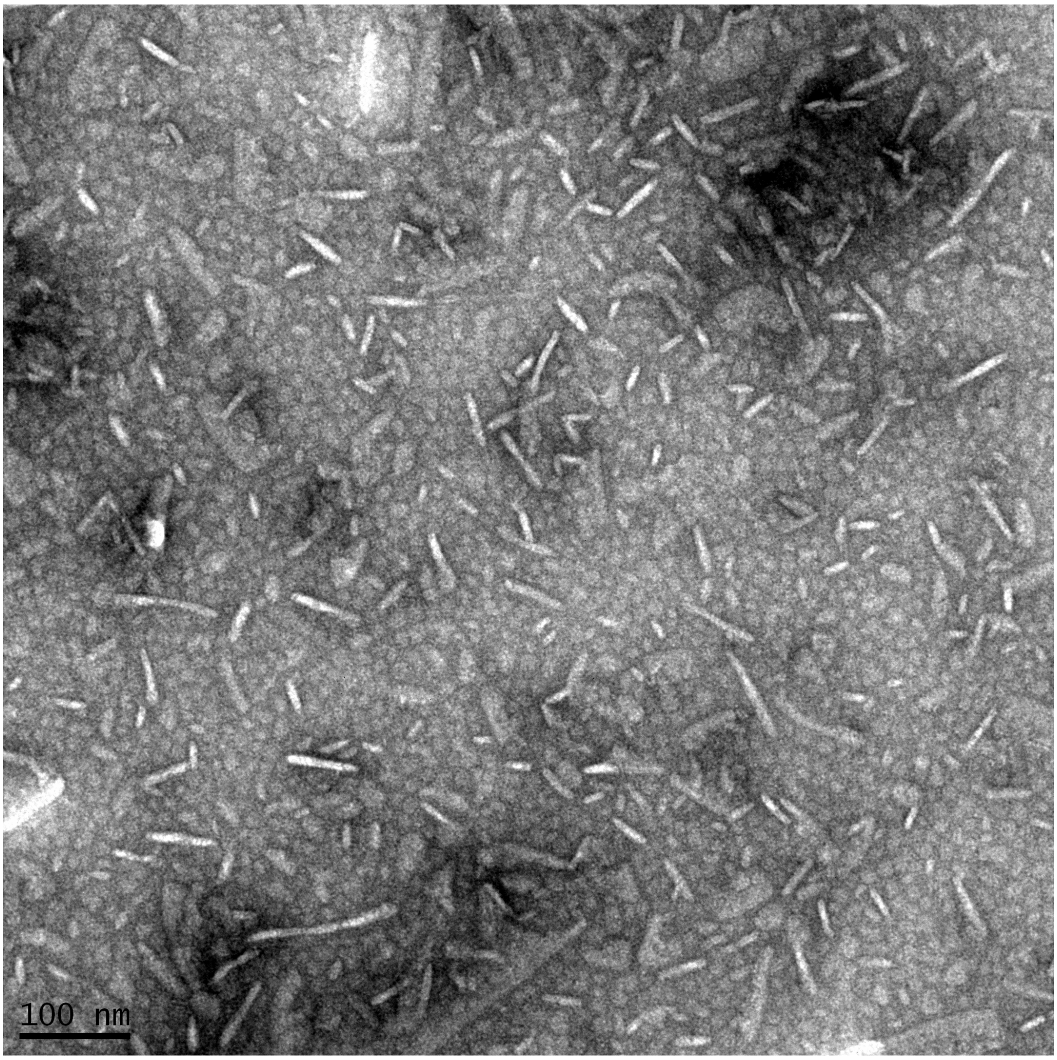

Lipids + Hsp12

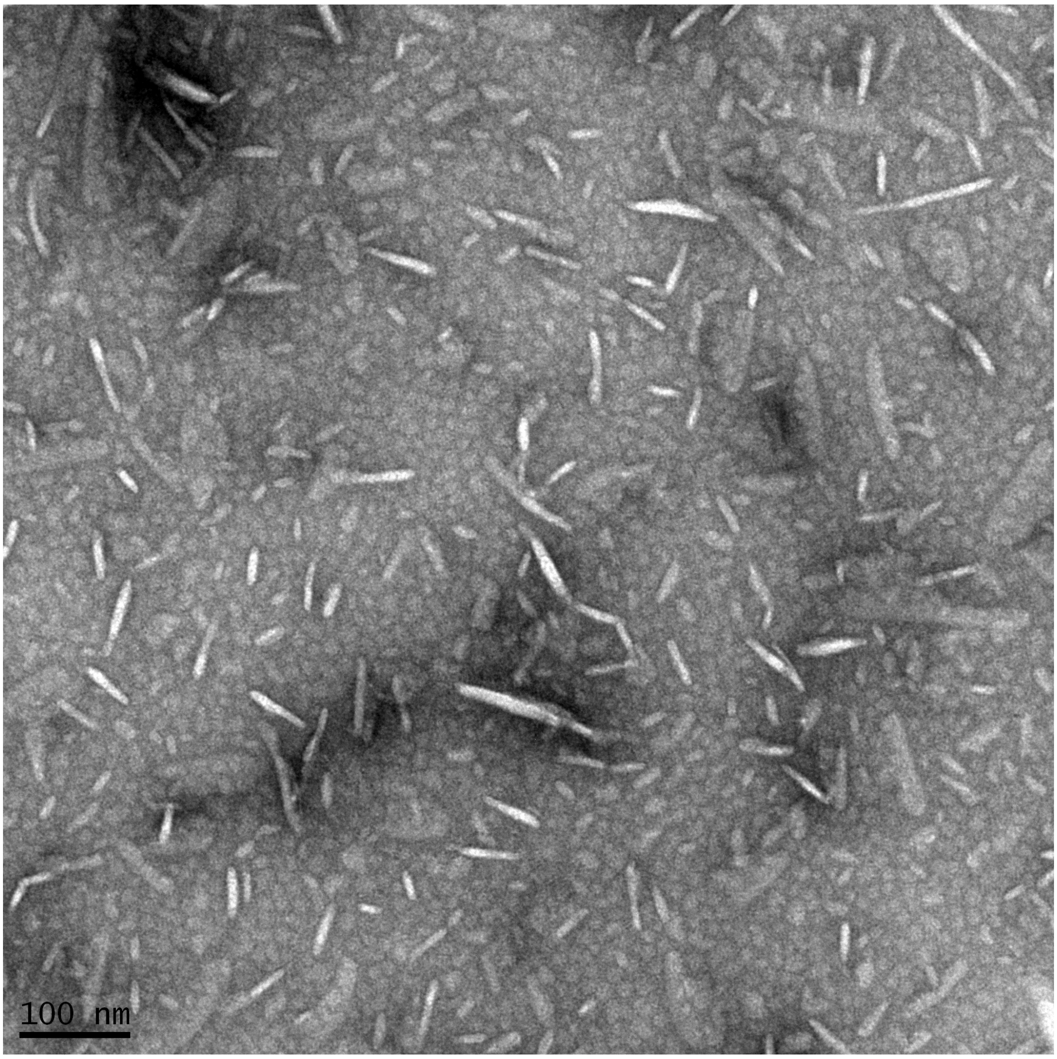

Lipids + Hsp12

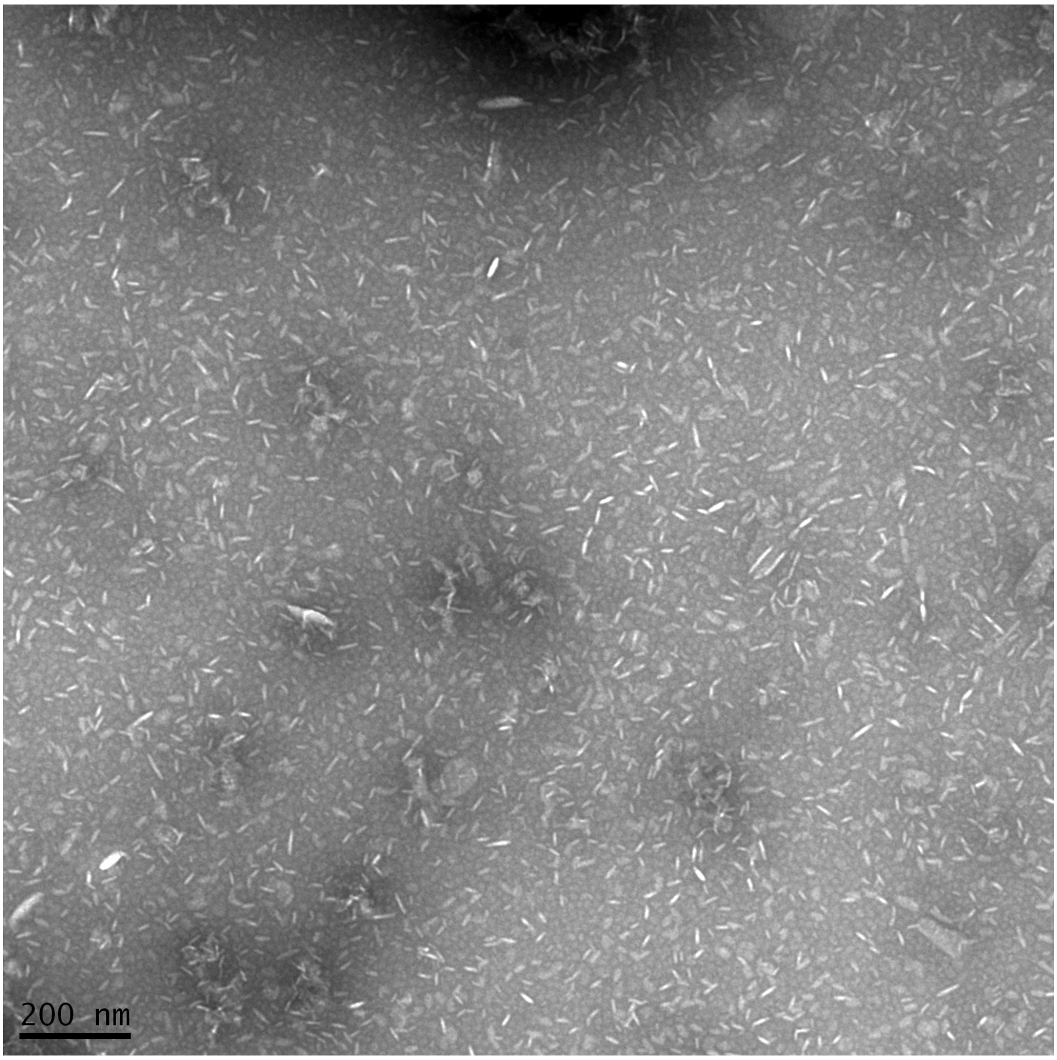

Supplement: Figure 4—source data 2. [file elife-38337-fig4-data2.pdf]
