# Supplementary material for: Synergy between the small intrinsically disordered protein Hsp12 and trehalose sustain viability after severe desiccation
Source: eLife. 2018 Jul 16;7:e38337. doi: 10.7554/eLife.38337 (PMC6054528; doi:10.7554/eLife.38337)
Supplement: Supplementary file 2. — (A) Three different groups where identified from our SGA - tps1∆ screen. (1) gene deletions that were synthetic lethal with tps1∆: failed to grow completely. (2) gene deletions that lead to desiccation sensitivity with tps1∆, and (3) gene deletions that allowed tps1∆ to grow after 30 days of drying: suppressors. List of genes attached as 1–3. (B) Breakdown of desiccation sensitive tps1Δ double mutants into categories based on cellular function. Each desiccation sensitive double mutant was placed into a category based on their cellular function (GO). [file elife-38337-supp2.docx]

Table Supplement 1. Synthetic Genetic Array Desiccation Screen – *tps1∆*

# A.

|  | Number of Hits | Total Genes |
| --- | --- | --- |
| Synthetic Lethal with *tps1∆* | 178 | 4700 |
| Desiccation Sensitive with *tps1∆* | 174 | 4700 |
| Desiccation Tolerant suppressors of *tps1∆* after 30 Day Dry | 121 | 4700 |

**B.**


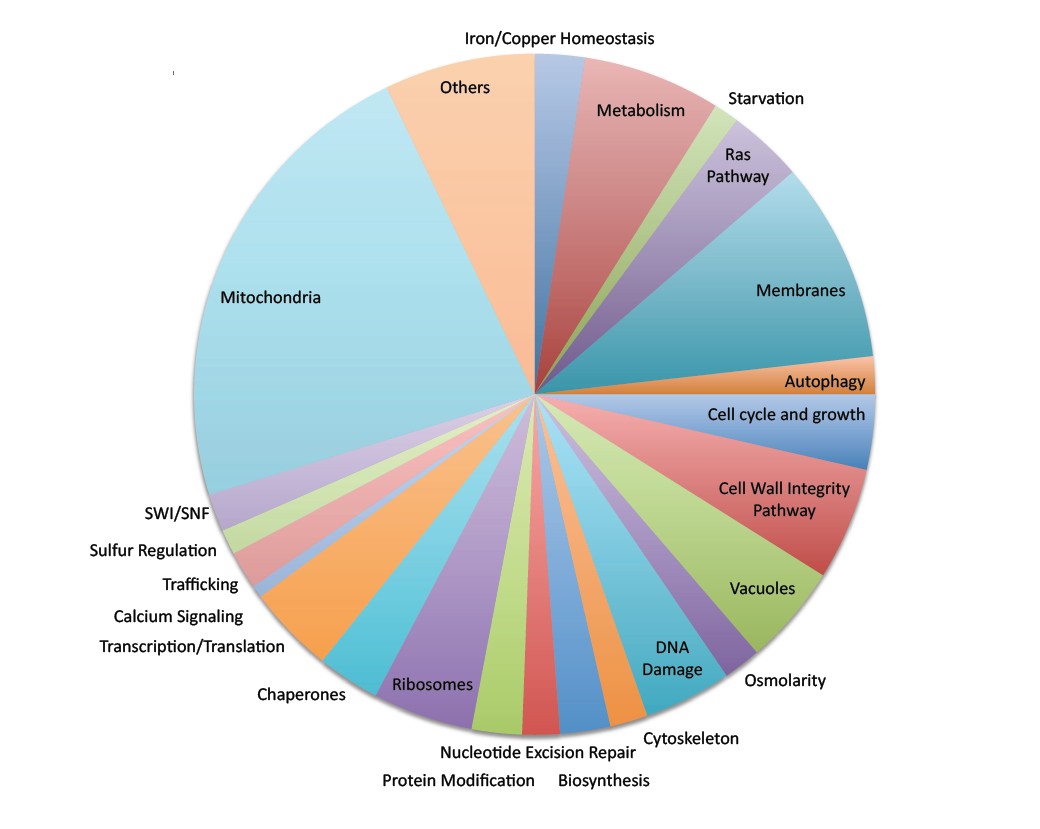


1. Synthetic Lethality with *tps1∆* - SGA

| **Strain** | ***Standard Name*** | **Strain** | ***Standard Name*** | **Strain** | ***Standard Name*** | **Strain** | ***Standard Name*** |
| --- | --- | --- | --- | --- | --- | --- | --- |
| YBR278W | *DPB3* | YMR154C | RIM13 | YML073C | YL16A | YDR419W | *RAD30* |
| YBR279W | *PAF1* | YBR191W | RPL21A | YNL055C | POR1 | YEL024W | *RIP1* |
| YBR282W | *MRPL27* | YLR370C | ARC18 | YNL284C | MRPL10 | YEL027W | *VMA3* |
| YCR020W-B | *HTL1* | YLR382C | NAM2 | YPR133W-A | TOM5 | YEL029C | *BUD16* |
| YCR024C | *SLM5* | YJR118C | ILM1 | YAL044C | GCV3 | YEL043W |  |
| YCR044C | *PER1* | YJR139C | HOM6 | YAL039C | CYC3 | YEL044W | *IES6* |
| YCR063W | *BUD31* | YJL006C | CTK2 | YAL013W | DEP1 | YEL051W | *VMA8* |
| YCR071C | *IMG2* | YJR030C | RBH2 | YAL002W | VPS8 | YER050C | *RSM18* |
| YCR081W | *SRB8* | YJR033C | RAV1 | YBL016W | *FUS3* | YER044C | *BUD18* |
| YDR363W-A | *SEM1* | YJR035W | RAD26 | YBL038W | *MRPL16* | YFL036W | *RPO41* |
| YFR040W | *SAP155* | YJR036C | HUL4 | YBL063W | *KIP1* | YFL053W | *DAK2* |
| YGR219W |  | YJR040W | GEF1 | YBL066C | *SEF1* | YFR001W | *LOC1* |
| YGR229C | *SMI1* | YJR060W | CBF1 | YBL101C | *ECM21* | YFR010W | *UBP6* |
| YGR237C |  | YJR066W | TOR1 | YBR015C | *TTP1* | YGL016W | *PDR6* |
| YGR270W | *YTA7* | YJL136C | RPS25B | YBR042C | *CST26* | YGL025C | *PGD1* |
| YGR275W | *RTT102* | YJL165C | HAL5 | YBR065C | *ECM2* | YGL058W | *RAD6* |
| YGR285C | *ZUO1* | YJL175W |  | YBR094W | *PBY1* | YGL124C | *MON1* |
| YHR039C-B | *VMA10* | YJL184W | GON7 | YBR098W | *MMS4* | YGL136C | *MRM2* |
| YKR035W-A | *DID2* | YJL189W | RPL39 | YBR188C | *NTC20* | YGL151W | *NUT1* |
| YKR082W | NUP133 | YFR025C | HIS2 | YBR221C | *PDB1* | YGL244W | *RTF1* |
| YLL018C-A | COX19 | YJL101C | GSH1 | YBR262C | *AIM5* | YGR003W | *CUL3* |
| YNR050C | LYS9 | YKR085C | MRPL20 | YCL032W | *STE50* | YGR020C | *VMA7* |
| YNR068C | BUL3 | YLR439W | MRPL4 | YCL037C | *SRO9* | YGR055W | *MUP1* |
| YER106W | MAM1 | YLR442C | SIR3 | YCR007C |  | YGR101W | *PCP1* |
| YER108C | FLO8 | YNR052C | POP2 | YCL039W | *GID7* | YGR104C | *SRB5* |
| YER141W | COX15 | YPL268W | PLC1 | YCL063W | *VAC17* | YGR122W |  |
| YER145C | FTR1 | YPR067W | ISA2 | YDL033C | *SLM3* | YGR171C | *MSM1* |
| YER151C | UBP3 | YBR131W | CCZ1 | YDL045W-A | *MRP10* | YGR215W | *RSM27* |
| YER154W | OXA1 | YBR289W | SNF5 | YDL069C | *CBS1* | YGR217W | *CCH1* |
| YER161C | SPT2 | YCR028C-A | RIM1 | YDL072C | *YET3* | YHL027W | *RIM101* |
| YER177W | BMH1 | YCR046C | IMG1 | YDL159W | *STE7* | YHL007C | *STE20* |
| YMR058W | FET3 | YCR047C | BUD23 | YDL185W | *VMA1* | YHR121W | *LSM12* |
| YMR066W | *SOV1* | YCR084C | TUP1 | YDR027C | *VPS54* | YHR132C | *ECM14* |
| YMR077C | *VSP20* | YNL052W | COX5A | YDR123C | *INO2* | YIL040W | *APQ12* |
| YOL086C | *ADH1* | YNL073W | MSK1 | YDR175C | *RSM24* | YIL148W | *RPL40A* |
| YOL115W | TRF4 | YNL107W | YAF9 | YDR226W | *ADK1* | YIL149C | *MLP2* |
| YOL129W | *VPS64* | YDR512C | EMI1 | YDR227W | *SIR4* | YIL153W | *RRD1* |
| YER092W | IES5 | YFL001W | DEG1 | YDR237W | *MRPL7* |  | |
| YHL025W | SNF6 | YFL013C | IES1 | YDR279W | *RNH202* |  |  |
| YHR064C | SSZ1 | YGR252W | GCN5 | YDR293C | *SSD1* |  |  |
| YHR168W | MTG2 | YGR255C | COQ6 | YDR295C | *HDA2* |  |  |
| YOR096W | RPS7A | YGR257C | MTM1 | YDR296W | *MHR1* |  |  |
| YMR097C | MTG1 | YIR023W | DAL81 | YDR332W | *IRC3* |  |  |
| YMR135C | GID8 | YJR055W | HIT1 | YDR335W | *MSN5* |  |  |
| YHR005C | GPA1 | YNL138W | SRV2 | YDR388W | *RVS167* |  |  |
| YHR041C | SRB2 | YOR270C | VPH1 | YDR389W | *SAC7* |  |  |
| YHR067W | HTD2 | YPL078C | ATP4 | YDR405W | *MRP20* |  |  |

1. Desiccation Sensitive with *tps1∆* - 6 Day Dry - SGA

| **Strain** | ***Standard Name*** | **Strain** | ***Standard Name*** | **Strain** | ***Standard Name*** | **Strain** | ***Standard Name*** |
| --- | --- | --- | --- | --- | --- | --- | --- |
| **YFL014W** | ***HSP12*** | YJR052W | *RAD7* | YKL137W | *CMC1* | YNR002C | *ATO2* |
| YBR295W | *PCA1* | YJR061W |  | YKL150W | *MCR1* | YNR039C | *ZRG17* |
| YBR297W | *MAL33* | YFL033C | *RIM15* | YKL159C | *RCN1* | YNR047W | *FPK1* |
| YCR026C | *NPP1* | YAL008W | *FUN14* | YKL161C | *KDX1* | YOR061W | *CKA2* |
| YCR027C | *RHB1* | YBL001C | *ECM15* | YKL170W | *MRPL38* | YOR065W | *CYT1* |
| YGR256W | *GND2* | YBR019C | *GAL10* | YKL171W | *NNK1* | YOR086C | *TCB1* |
| YIR021W | *MRS1* | YBR037C | *SCO1* | YKL175W | *ZRT3* | YOR107W | RGS2 |
| YIR023W | *DAL81* | YBR203W | *COS111* | YKL207W | *EMC3* | YOR115C | TRS33 |
| YIR031C | *DAL7* | YBR215W | *HPC2* | YKR003W | *OSH6* | YOR118W | RTC5 |
| YIR033W | *MGA2* | YBR227C | *MCX1* | YKR055W | *RHO4* | YOR149C | *SMP3* |
| YIR038C | *GTT1* | YCR009C | *RVS161* | YKR059W | *TIF1* | YOR161C | PNS1 |
| YLR423C | *ATG17* | YDL107W | *MSS2* | YLL006W | *MMM1* | YOR166C | SWT1 |
| YLR425W | *TUS1/SOP10* | YDR148C | *KGD2* | YLL014W | *EMC6* | YOR178C | *GAC1* |
| YLR447C | *VMA6* | YDR194C | *MSS116* | YLL019C | *KNS1* | YOR182C | *RPS30B* |
| YER118C | *SSU81* | YDR197W | *CBS2* | YLL026W | *HSP104* | YOR191W | ULS1 |
| YER128W | *VFA1* | YDR204W | *COQ4* | YLL040C | *VPS13* | YOR231W | *MKK1* |
| YER153C | *PET122* | YDR350C | *TCM10* | YLL043W | *FPS1* | YOL015W | *IRC10* |
| YER155C | *BEM2* | YDR375C | *BCS1* | YLL051C | *FRE6* | YOL083W | *ATG34* |
| YER167W | *BCK2* | YER030W | *CHZ1* | YLR025W | *SNF7* | YOL084W | *PHM7* |
| YMR070W | *MOT3* | YFL016C | *MDJ1* | YLR006C | *SSK1* | YPL274W | *SAM3* |
| YOL095C | *HMI1* | YGL107C | *RMD9* | YLR064W | *PER33* | YPL273W | *SAM4* |
| YOL096C | *COQ3* | YGL115W | *SNF4* | YLR079W | *SIC1* | YPL249C | *GYP5* |
| YER078C | *ICP55* | YGL135W | *RPL1B* | YLR080W | *EMP46* | YPL193W | *RSA1* |
| YER008C | *SEC3* | YGL203C | *KEX1* | YLR083C | *EMP70* | YPL178W | *CBC2* |
| YDR424C | *DYN2* | YGL213C | *SKI8* | YLR093C | *NYV1* | YPL105C | *SYH1* |
| YER090W | *TRP2* | YGL234W | *ADE5,7* | YLR114C | *AVL9* | YPL096W | *PNG1* |
| YEL011W | *GLC3* | YGR078C | *PAC10* | YLR134W | *PDC5* | YPL089C | *RLM1* |
| YLR034C | *SMF3* | YGR105W | *VMA21* | YLR375W | *STP3* | YPL057C | *SUR1* |
| YMR315W |  | YGR183C | *QCR9* | YMR035W | *IMP2* | YPL049C | *DIG1* |
| YMR316C-A |  | YHL038C | *CBP2* | YMR256C | *COX7* | YPR003C |  |
| YML095C | *RAD10* | YHL023C | *NPR3* | YNL339C | *YRF1-6* | YPR004C | *AIM25* |
| YML108C |  | YHR010W | *RPL27A* | YNL330C | *RPD3* | YPR124W | *CTR1* |
| YML110C | *COQ5* | YHR011W | *DIA4* | YNL329C | *PEX6* | YPR194C | *OPT2* |
| YMR099C |  | YHR016C | *YSC84* | YNL233W | *BN14* | YJR004C | *SAG1* |
| YER063W | *THO1* | YHR021C | *RPS27B* | YNL229C | *URE2* |  | |
| YER066W | *RRT13* | YIL013C | *PDR11* | YNL225C | *CNM67* |  |  |
| YLR246W | *ERF2* | YIL162W | *SUC2* | YNL218W | *MGS1* |  |  |
| YLR346C |  | YJL168C | *EZL1* | YNL162W | *RPL41A* |  |  |
| YPL217C | *BMS1* | YJL046W | *AIM22* | YNL145W | *MFA2* |  |  |
| YPL224C | *MMT2* | YKL002W | DID4 | YNL139C | *THO2* |  |  |
| YCL023C |  | *YKL037W* | *AIM26* | YNL144C |  |  |  |
| YJR087W |  | YKL048C | *ELM1* | YNL098C | *RAS2* |  |  |
| YJR134C | *SGM1* | YKL055C | *OAR1* | YNL091W | NST1 |  |  |
| YML072C | *TCB3* | YKL101W | *HSL1* | YNL077W | APJ1 |  |  |
| YML085C | *TUB1* | YKL134C | *OCT1.* | YNL054W | *VAC7* |  |  |
| YJL005W | *CYR1* | YKL146W | *AVT3* | YNL001W | *DOM34* |  |  |
| YJL028W |  | YKL148C | *SDH1* | YNR001C | *CIT1* |  |  |

1. Desiccation Tolerant Suppressors of *tps1∆* - 30 Day Dry - SGA

| **Strain** | ***Standard Name*** | **Strain** | ***Standard Name*** | **Strain** | ***Standard Name*** |
| --- | --- | --- | --- | --- | --- |
| YER093C-A | *AIM11* | YDR108W | *GSG1* | YIR013C | *GAT4* |
| YMR142C | *RPL14B* | YDR159W | *SAC3* | YJL170C | *ASG7* |
| YMR319C | *FET4* | YDR162C | *NBP2* | YJL165C | *HAL5* |
| YML094W | *GIM5* | YDR173C | *ARG82* | YJL079C | *PRY1* |
| YMR106C | *YKU80* | YDR207C | *UME6* | YJL038C | *LOH1* |
| YMR116C | *ASC1* | YDR231C | *COX2* | YJR092W | *BUD4* |
| YER031C | *YPT8* | YDR264C | *AKR1* | YKL096W | *CWP1* |
| YER046W | *SPO73* | YDR320C | *SWA2* | YKL103C | *APE1* |
| YGR204W | *ADE3* | YDR360W | *OPI7* | YKL114C | *APN1* |
| YHR119W | *YTX1* | YDR369C | *XRS2* | YKL119C | VPH2 |
| YJR121W | *ATP2* | YDR432W | *NPL3* | YKL126W | YPK1 |
| YJR140C | *HIR3* | YDR441C | *APT2* | YKL127W | PGM1 |
| YAL054C | *ACS1* | YDR457W | *TOM1* | YKL185W | ASH1 |
| YJR010W | *MET4* | YDR511W | *ACN9* | YKL187C | FAT3 |
| YJR063W | *RPA12* | YDR524C | *AGE1* | YKL197C | PEX1 |
| YJR066W | *TOR1* | YEL007W | *MIT1* | YKR007W | MEH1 |
| YGR239C | *PEX21* | YEL052W | *AFG1* | YLL001W | DNM1 |
| YJL137C | *GLG2* | YER005W | *YND1* | YLL021W | SPA2 |
| YBR083W | *TEC1* | YFL011W | *HXT10* | YLL052C | AQY2 |
| YBR084C-A | *RPL19A* | YFL030W | *AGX1* | YLR068W | FYV7 |
| YBR125C | *PTC4* | YFL055W | *AGP3* | YLR070C | XYL2 |
| YBR168W | *PEX32* | YFL008W | *SMC1* | YLR074C | BUD20 |
| YNL079C | *TPM1* | YGL006W | *PMC1* | YLR121C | YPS3 |
| YNL080C | *EOS1* | YGL028C | *SCW11* | YLR172C | DPH5 |
| YOR265W | *RBL2* | YGL110C | *CUE3* | YLR191W | PEX13 |
| YNL059C | *ARP5* | YGL153W | *PEX14* | YLR206W | ENT2 |
| YOL148C | *SPT20* | YGL216W | *KIP3* | YLR216C | CPR6 |
| YAL059W | *SIM1* | YGL227W | *VID30* | YLR248W | RCK2 |
| YAL035W | *FUN12* | YGL229C | *SAP4* |  | |
| YAL011W | *SWC3* | YGL236C | *MTO1* |  |  |
| YBL027W | *RPL19B* | YGR007C | *MUQ1* |  |  |
| YBL006C | *LDB7* | YGR016W |  |  |  |
| YBL021C | *HAP3* | YGR025W |  |  |  |
| YBL045C | *COR1* | YGR059W | *SPR3* |  |  |
| YBL080C | *PET112* | YGR141W | *VPS62* |  |  |
| YBL089W | *AVT5* | YGR144W | *THI4* |  |  |
| YBR136W | *ESR1* | YGR157W | *CHO2* |  |  |
| YBR229C | *ROT2* | YHL044W |  |  |  |
| YDL009C |  | YHR109W | *CTM1* |  |  |
| YDL021W | *GPM2* | YHR113W | *APE4* |  |  |
| YDL038C |  | YHR115C | *DMA1* |  |  |
| YDL116W | *NUP84* | YHR132C | *ECM14* |  |  |
| YDL137W | *ARF2* | YIL011W | *TIR3* |  |  |
| YDL191W | *RPL35A* | YIL039W | *TED1* |  |  |
| YDL215C | *GDH2* | YIL088C | *AVT7* |  |  |
| YDR024W | *FYV1* | YIL090W | *ICE2* |  |  |
| YDR025W | *RPS11A* | YIL095W | *PRK1* |  |  |
