# Supplementary material for: Synergy between the small intrinsically disordered protein Hsp12 and trehalose sustain viability after severe desiccation
Source: eLife. 2018 Jul 16;7:e38337. doi: 10.7554/eLife.38337 (PMC6054528; doi:10.7554/eLife.38337)
Supplement: Supplementary file 3. — Strains used in this study. [file elife-38337-supp3.docx]

Table Supplement 2. Strain Table

| **Stain List** |  |  |
| --- | --- | --- |
| BY4742 | Wild type | MATα his3Δ1 leu2Δ0 lys2Δ0 ura3Δ0 |
| HTY104 | *tps1∆* | MATα his3Δ1 leu2Δ0 lys2Δ0 ura3Δ0 tps1::G418 |
| HTY11 | *hsp12∆* | MATα his3Δ1 leu2Δ0 lys2Δ0 ura3Δ0 hsp12::G418 |
| HTY176 | *tps1∆hsp12∆* | MATα his3Δ1 leu2Δ0 lys2Δ0 ura3Δ0 tps1::HYG hsp12::G418 |
| HTY14 | *stf2∆* | MATα his3Δ1 leu2Δ0 lys2Δ0 ura3Δ0 stf2::G418 |
| HTY179 | *tps1∆stf2∆* | MATα his3Δ1 leu2Δ0 lys2Δ0 ura3Δ0 tps1::HYG stf2::G418 |
| HTY195 | *8X∆:*  *hsp12Δgre1Δsip18Δstf 2Δnop6Δybr016wΔyjl1 44wΔynl190wΔ* | hsp12::GFP, gre1::GFP, sip18::GFP, stf2::GFP, ybr016w::GFP,  yjl144w::GFP, ynl190w::GFP, nop6::HYG |
| **Transporter Strains** |  |  |
| HTY121 | *nth1∆* | MATα his3Δ1 leu2Δ0 lys2Δ0 ura3Δ0 nth1::G418 |
| HTY164 | *nth1∆ AGT1* | MATα his3Δ1 leu2Δ0 lys2Δ0 ura3Δ0 nth1::G418 can1::AGT1 |
| **Prion Strains** |  |  |
| HTY92 | [*PSI+*] | Mat α ade1-14 trp1-289 his3∆-200 ura3-52 leu2-3,112 (ADE+) |
| HTY187 | [*PSI+*] *hsp12∆* | Mat α ade1-14 trp1-289 his3∆-200 ura3-52 leu2-3,112 (ADE+) hsp12::HYG |
| HTY120 | [*PSI+*] *tps1∆* | Mat α ade1-14 trp1-289 his3∆-200 ura3-52 leu2-3,112 (ADE+) tps1::G418 |
| HTY188 | [*PSI+*] *tps1∆ hsp12∆* | Mat α ade1-14 trp1-289 his3∆-200 ura3-52 leu2-3,112 (ADE+) tps1::G418 hsp12::HYG |
| HTY82 | [*GAR+*] | Mat a can1-100 his3-11,15 leu2-2,112 trp1-1 ura3-1, ade2-1 (GlucosamineR) |
| HTY189 | [*GAR+*] *hsp12∆* | Mat a can1-100 his3-11,15 leu2-2,112 trp1-1 ura3-1, ade2-1 (GlucosamineR) hsp12::HYG |
| HTY115 | [*GAR+*] tps1*∆* | Mat a can1-100 his3-11,15 leu2-2,112 trp1-1 ura3-1, ade2-1 (GlucosamineR) tps1::G418 |
| HTY190 | [*GAR+*] *tps1∆hsp12∆* | Mat a can1-100 his3-11,15 leu2-2,112 trp1-1 ura3-1, ade2-1 (GlucosamineR) tps1::NAT hsp12::HYG |
